# Workshop on Bridging Satellite Climate Data Gaps

**DOI:** 10.6028/jres.116.002

**Published:** 2011-02-01

**Authors:** Catherine Cooksey, Raju Datla

**Affiliations:** Naional Institute of Standards and Technology, Gaithersburg, MD 20899

**Keywords:** calibration, climate data gaps, remote sensing satellite data, SI traceability

## Abstract

Detecting the small signals of climate change for the most essential climate variables requires that satellite sensors make highly accurate and consistent measurements. Data gaps in the time series (such as gaps resulting from launch delay or failure) and inconsistencies in radiometric scales between satellites undermine the credibility of fundamental climate data records, and can lead to erroneous analysis in climate change detection. To address these issues, leading experts in Earth observations from National Aeronautics and Space Administration (NASA), National Oceanic and Atmospheric Adminstration (NOAA), United States Geological Survey (USGS), and academia assembled at the National Institute of Standards and Technology on December 10, 2009 for a workshop to prioritize strategies for bridging and mitigating data gaps in the climate record. This paper summarizes the priorities for ensuring data continuity of variables relevant to climate change in the areas of atmosphere, land, and ocean measurements and the recommendations made at the workshop for overcoming planned and unplanned gaps in the climate record.

## 1. Introduction

On December 10, 2009, the Optical Technology Division of the Physics Laboratory of the National Institute of Standards and Technology (NIST) held a workshop for experts in satellite remote sensing from the National Aeronautics and Space Administration (NASA), the National Oceanic and Atmospheric Administration (NOAA), the United States Geological Survey (USGS), and other government and non-government organizations to discuss strategies for overcoming data gaps that may occur in satellite sensor observations. Developing and implementing effective strategies requires considerable forethought because the requirements of high accuracy for observing climate change are applicable through any data gap and are critical for maintaining irrefutable climate records.

Drs. Raju Datla of the Optical Technology Division at NIST, Changyong Cao of the Center for Satellite Applications and Research (STAR) of NOAA, and James Butler of NASA Goddard Space Flight Center organized the workshop on behalf of their respective agencies to address the problem and recommend strategies for future planning. The one-day workshop began with invited talks in the morning session and concluded in the afternoon with discussions and consensus recommendations.

## 2. Highlights From the Morning Session

Dr. Katharine Gebbie, the Director of the Physics Laboratory, welcomed everyone on behalf of the NIST Director, Dr. Patrick Gallagher. Reflecting on the purpose of the meeting, she noted that the need for acquiring highly accurate satellite measurements from different instruments on different satellites in different wavelength regions over very long periods of time is an important issue because it is upon these measurements that critical policy decisions will be made regarding climate change.

**Fig. 1:**
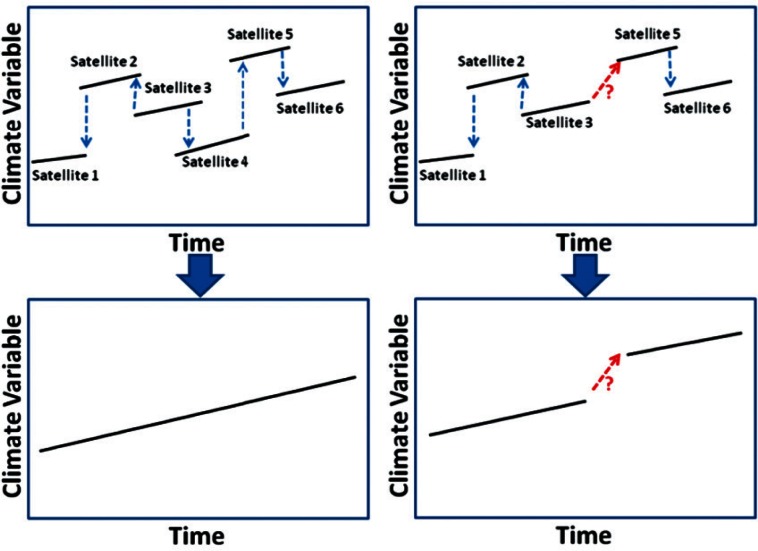
An illustration of the risk for disruption in the acquisition of data by satellites. In the left panel, the temporal overlap of a series of satellite observations enables reconciliation of the data over the entire time period. Whereas, the absence of data from Satellite 4 in the right panel hampers reconciliation between data from the first three time series (Satellites 1, 2, and 3) and the last two (Satellites 5 and 6).

Next, Dr. Gerald Fraser, Chief of the Optical Technology Division, stated the goal of the meeting. He referred to the recent Government Accountability Office (GAO), [1] Office of Inspector General (OIG), [2,3] and National Research Council (NRC) [4] reports that highlight concerns about possible disruptions in data records tied to the NPOESS^1^ and NPP^2^ satellite programs. For example, the OIG report, *Top Management Challenges Facing the Department of Commerce, Final Report No. OIG-19384*, states that such a disruption “could have serious consequences for the safety and security of the nation.” To illustrate this point, Fraser showed the example in Fig. 1 in which the overlapping time series of satellite observations in the left panel enable reconciliation of the data records; whereas, the failure of a sensor or satellite in the series in the right panel makes assembling the data record tenuous. Therefore, he challenged the participants to identify strategies to address the data gaps that are inevitable given the lack of redundancy in these satellite programs and others.

The remaining talks from the morning session laid the foundation for the afternoon discussion sessions. The talks highlighted the challenges of measuring the small signatures expected for climate change against the larger daily and yearly variations due to weather and cyclical climate behaviors associated with events such as El Niño. A range of measurement topics related to measuring climate change from space, including the current state of measurement capabilities, sensor calibration, SI traceability, future plans for improved data acquisition, and NIST’s current involvement in these efforts was covered. These topics are relevant to the workshop’s goals because maintaining accurate and irrefutable climate records necessarily involves methods for bridging and mitigating gaps while preserving low measurement uncertainties.

### 2.1 Transfer Standards—NIST’s Role

Calibration, and ultimately the SI traceability, of satellite sensors is dependent on the transfer of measurement scales to the sensor. Three of the morning’s speakers described their experience with transfer standards at different stages in the calibration process: development of standards and calibration methods, pre-launch and onboard calibrations in retrospect, and planning for future pre-launch and onboard calibrations.

Fuzhong Weng of NOAA spoke about the microwave sensors, their current calibration methodologies, and the use of their data for Numerical Weather Prediction (NWP). Microwave sensors observe climate parameters important for maintaining atmospheric temperature, sea-surface temperature (SST), and water-vapor records. Weng explained that each microwave sensor is different, and acknowledged that there is currently no pre-launch effort sufficient to establish SI traceability. Furthermore, Weng said that there is lack of community effort to define traceability and to develop needed reference standards, and he encouraged the microwave community to work with NIST to develop needed standards, highlighting a particular need for better characterization of antenna patterns.^3^ These efforts are critical for reducing the uncertainties of microwave measurements to the levels required for climate monitoring.

In contrast to the microwave region, the development and implementation of transfer standards for the calibration of sensors in the reflected solar region is relatively mature. Drs. Jack Xiong and James Butler, both of NASA Goddard, co-presented a talk titled, “MODIS lessons: NIST role in sensor calibration and inter-comparison.” MODIS^4^ (Moderate Resolution Imaging Spectroradiometer) detects narrow bands of visible and infrared radiation from the Earth with varying degrees of spatial resolution. Xiong explained that despite MODIS’s comprehensive pre-calibration and characterization process and its onboard calibration features, which include a solar diffuser calibrated by the vendor using a NIST reference standard, on-orbit comparison of measurements by the solar diffuser with other satellite sensors, and a solar diffuser stability monitor, the uncertainties were not small enough for climate monitoring. Xiong suggested that the lack of a system-level calibration of the solar diffuser and multiple calibration transfer steps resulted in poorly estimated uncertainty values. Based on this experience, Xiong endorsed reducing transfer steps and performing system-level calibrations, which include all planned measurement geometries, to aid diagnosis of on-orbit temporal changes in the uniformity of the solar diffuser and minimize unnecessary increases in the measurement uncertainties.

**Fig. 2:**
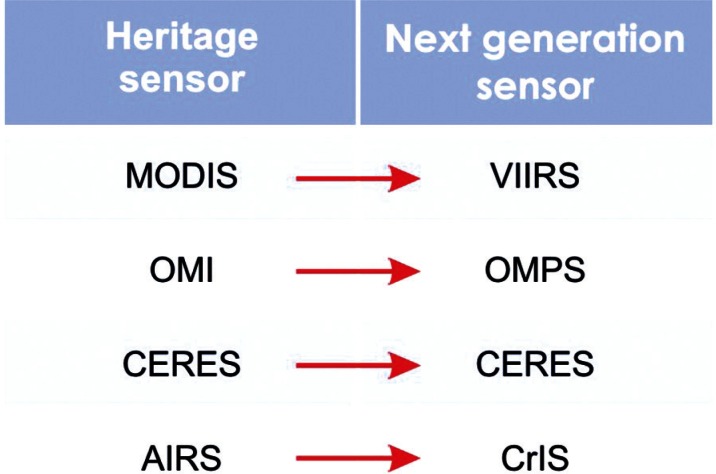
Transitioning from EOS to NPOESS.^5^ The table shows the correspondence between satellite sensors employed currently by EOS (heritage sensors) and the next generation sensors that will continue the scientific data record under NPOESS. MODIS: Moderate Resolution Imaging Spectroradiometer; VIIRS: Visible/Infrared Imager/Radiometer Suite; OMI: Ozone Monitoring Instrument; OMPS: Ozone Mapping and Profiler Suite; CERES: Clouds and the Earth’s Radiant Energy System; AIRS: Atmospheric InfraRed Sounder; CrIS: Cross Track Infrared Sounder.

Butler focused his remarks on the evolution of NASA’s collaboration with NIST as NASA has shifted from Earth Observing System (EOS)^5^ satellite sensors to the next generation sensors of NPOESS. The charter for the NPOESS Preparatory Project (NPP) charged NASA to continue the scientific data records started by EOS. Thus, NASA and NIST are building on their experiences calibrating and characterizing MODIS in preparation for the next generation sensor, the Visible Infrared Imager Radiometer Suite (VIIRS) (Fig. 2). As an example, Butler described the NIST-built Flat Plate Integrator (FPI), a white diffuser that can be illuminated by tunable lasers or a Xenon arc source through fiber-optic feeds, which will be used in the (VIIRS) test chamber. The FPI will act as a tunable source for characterizing the sensor at the system level after the instrument is installed on the spacecraft, and will enable validation of the reflective solar band gains of the sensor, an end-to-end calibration of the solar diffuser Earth view, and measurement of the out-of-band and in-band relative spectral response of the sensor in thermal vacuum.^6^ These activities represent the first time that calibrations and characterization of this type will be done for a sensor in the solar reflective region. Butler also mentioned a similar effort to validate the VIIRS blackbody using NIST’s Thermal Infrared Transfer Radiometer (TXR). He described these collaborations as positive and crucial for addressing future measurement issues such as data gaps.

## 2.2 Moon as the Celestial Transfer Standard

Dr. Thomas Stone of USGS described his agency’s efforts to calibrate the Moon in the reflected solar wavelength range for use as a reliable standard for on-orbit radiometric calibration of solar-band instruments. This capability is important for quantifying the change in sensor performance between pre-launch calibration and on-orbit operation. Current best practices for on-orbit calibration involve measurement comparisons against well-characterized ground sites (i.e., vicarious calibration) or using an on-board stability monitor to measure the degradation of the reflectance of the solar diffuser. However, the lower limit of uncertainty achievable with these methods is two to three percent, which is too large for long-term monitoring of climate change.

As a first step towards establishing a celestial standard, the USGS has developed an analytical model to account for variations in the brightness of the Moon due to phase and libration based on observations made from Flagstaff, AZ using the Robotic Lunar Observatory (ROLO)[5]. The advantage of this method over the current best practices is the exceptional stability of the reference source: the Moon has existed for billions of years and lacks dynamic forces and properties such as plate tectonics, an atmosphere, vegetation, and significant human activity. The value of the ROLO model has been recognized by the operational programs in the United States and in other countries. For example, NOAA has instituted regular, dedicated observations of the Moon by the GOES satellites and comparisons of the data with the model are currently being used, for research purposes, to improve the quality of the sensor’s radiometric measurements. Unfortunately, the absolute uncertainty of the irradiances derived from the ROLO model is 5 % to 10 %, and the SI-traceability of the values is poor. These deficiencies in the ROLO model limit the utility of the Moon as an absolute calibration reference for bridging gaps between missions.

Consequently, NIST, USGS, and others are developing a program called Lunar Spectral Irradiance and Radiance (LUSI) to establish the Moon as an absolute radiometric standard traceable to the *Système International d’Unités* (SI)[6]. The measurement objectives of LUSI are high accuracy, SI traceability, and continuous spectral coverage over the entire reflected solar wavelength range. Plans include a multi-year measurement campaign, with specialized instruments designed and calibrated by NIST. A ground-based instrument suite, which will undergo regular calibration, will be located at a premier astronomical observatory site and operate for several years to obtain the necessary time series of lunar measurements. A second instrument suite will be flown on a high-altitude balloon to acquire absolute lunar measurements above 99 % of the atmosphere. NIST will characterize and calibrate both sets of lunar radiometer instruments prior to deployment and perform pre- and post-flight calibrations for the balloon-based suite. The resulting lunar model will be based on the ROLO irradiance model,and aim to achieve an uncertainty of 0.5 % (*k* = 1).

### 2.3 Global Space-Based Inter-Calibration System (GSICS)

Dr. Mitch Goldberg of NOAA spoke about GSICS, an international collaboration launched by the World Meteorological Organization (WMO) and the Coordination Group for Meteorological Satellites (CGMS) to improve the comparability and accuracy of satellite measurements to meet the requirements for monitoring the climate and improving Numerical Weather Prediction (NWP). The goal of GSICS is to enhance the calibration and validation of satellite observations from space and to intercalibrate the critical components of the global observing system. Intercalibration of sensors within the satellite observing system will be accomplished using Simultaneous Nadir Overpasses (SNOs) to compare measurements acquired by two different, but similar, satellite sensors when viewing the same Earth scene at the same time. Goldberg noted that, while these intercomparisons are not currently traceable to the SI, he anticipates that NIST will play a critical role in GSICS by establishing SI-traceability and uncertainties, and improving the community’s understanding of the biases that become apparent during these intercomparisons. NIST, along with experts at NASA, has already provided guidance to GSICS, [7] and Goldberg believes NIST can contribute further to GSICS by improving calibrations with the use of SI-traceable transfer standards, establishing SI traceability for lunar absolute calibration, and supporting the calibration of airborne sensors used to validate satellite measurements.

### 2.4 Challenges for Interagency Cooperation

Within the United States, there are many agencies that participate in the development, implementation, and operation of satellites. Dr. Al Powell of NOAA’s Center for Satellite Applications and Research (STAR) described the challenges of accomplishing STAR’s mission to transfer new technologies and methodologies into routine operations because of a lack of a consistent, consolidated approach among the different agencies. Thus, NOAA is proposing the creation of a National Calibration Center that would enable NIST, NOAA, NASA, and other agencies to work cooperatively to identify and implement strategies to improve the calibration of operational satellite systems using expertise from across the three agencies.

NOAA’s National Climate Data Center (NCDC) faces a similar need for consistent, standard methods for calibration and intercalibration of operational satellite systems. Dr. John Bates of the NCDC spoke about its mission to archive and produce weather data and products. This effort typically includes archiving fundamental measurements, such as radiances, and creating climate data records (also known as retrieving climate variables). Recently, there has been increased interest in creating a climate information record in which the climate data records are applied to a particular problem, such as monitoring long-term changes in tropical cyclone intensity or assessing the cycles of water, energy, and carbon. All of these processes need to be calibrated and understood, and the uncertainties must be propagated from the observed radiances through the algorithms that retrieve the climate variables for the creation of a climate information record. However, none of this is possible without continuity of the data record, assembled from highly accurate measurements with time scales ranging from seasonal to inner-annual to decadal.

### 2.5 CLARREO—NASA Proposal for a SI-Traceable Climate Observatory in Space

Two of the morning’s speakers discussed the Climate Absolute Radiance and Refractory Observatory (CLARREO). This project was prioritized in the National Research Council’s report, “Earth Science and Applications from Space: National Imperatives for the Next Decade and Beyond,” [8] as a high priority activity to provide traceable, benchmark climate data for testing and validating models for operational climate forecasting.^7^

Keynote speaker, Dr. Bruce Wielicki of NASA Langley Research Center, explained that large spatial and temporal scales of the CLARREO’s measurements will average over the short-term dynamics of the climate and enable the possibility of observing the signatures of long-term climate change. This combined with the robust SI traceability of CLARREO’s calibrations and the high accuracy of its data will enable measurements made by CLARREO to serve as benchmarks. Table 1 lists the climate variables to be monitored by CLARREO and their corresponding observational information content. By employing these benchmarks for testing and improving climate forecasting, Wielicki said CLARREO mission will be able to accomplish its science objectives of studying anthrophogenic forcings and the response of the climate system.

In addition, CLARREO’s benchmark measurements will provide an opportunity to intercalibrate with other operational satellite sensors, such as CERES, MODIS, AIRS, and CrIS. Dr. Martin Mlynczak of NASA Langley Research Center described this aspect of the CLARREO mission, which will be critical for bridging gaps in satellite observations over the course of the next several decades. The proposed instrumentation for CLARREO will acquire solar reflective, infrared, and radio occultation measurements that are highly accurate, and these instruments will cover the entire spectrum from 5 m to 50 m with no gaps. There will be reference blackbodies on orbit in CLARREO that are monitored and have novel phase-transition cells tied to the freezing point of pure substances to keep the SI-traceable temperature setting, and a flight spare will be available to diagnose measurement problems that occur in the orbiting instrument. Thus, the traceability and accuracy achieved on orbit by CLARREO will surpass all other measurements of the reflected or emitted Earth radiation. In the visible spectral region, the goal is to achieve few tenths of a percent uncertain ty as opposed to the current level of 3 % to 5 %. The goal is challenging in the infrared region, a factor of two to three improvement in spectrally and spatially resolved brightness temperatures. Mlynczak added CLARREO is working closely with NIST in the development and implementation of calibration and characterization strategies to ensure a robust tie of the measurements to the SI.

### 2.6 Questions and Discussion From the Morning Session

A question was raised about strategies for deriving the retrieval products from CLARREO:

Martin Mlynczak clarified that the reflected solar and infrared optical sensors for CLARREO will generate data products different from those of typical weather or climate process satellites. The later satellite missions typically focus on instantaneous retrievals of a geophysical property, such as temperature profile or cloud height. In contrast, CLARREO will provide temporally and spatially averaged top-of-the-atmosphere spectral radiances for the infrared region and spectral reflectances for the reflected solar region. The only instantaneous geophysical parameters generated by CLARREO will be atmospheric temperature, which will be derived from radio occultation measurements.

The radiances and reflectances acquired by CLARREO will enable two new types of climate products. First, instantaneous radiance and reflectance measurements will be used to calibrate other satellite sensors and improve the ability of operational sensors to provide high accuracy decadal change data. These measurements will also be made available to the scientific user community for use in deriving instantaneous geophysical data products. Second, averaged infrared radiance and solar reflectance spectra will be used to derive fingerprints to monitor climate change and to establish its causes. These spectral fingerprints can be used to infer, through climate models, which climate-related physical parameters (e.g., atmospheric temperature, water vapor profile, clouds, radiative fluxes, surface albedo) are changing and how quickly they are changing. This fingerprinting approach can also be used to infer critical climate system feedbacks including the temperature lapse rate, water vapor, clouds, and surface albedo feedbacks.

*A question was raised about the way CLARREO will monitor the degradation of solar attenuators:*^8^

Bruce Wielicki answered that the solar attenuator will be measured by viewing the sun with the attenuator and viewing the Moon without the attenuator. These observations will be periodically repeated. Any degradation in the sensor system will be tracked by lunar views while degradation of the attenuator will be tracked by the observations of the sun. Neither method requires knowledge of absolute radiances of the Sun or Moon.

*A question was raised about the differences between the Moon and CLARREO as reference standards in space:*^9^

Thomas Stone noted that CLARREO offers unique opportunities to leverage the benefits of the Moon as an absolute long-term optical reference standard. For instance, CLARREO’s high accuracy sensor will provide opportunities for the intercalibration of satellites using simultaneous nadir overlap (SNO) observations of the Earth. CLARREO observations of the Moon can also be compared with the ROLO model or any future model developed to establish the Moon as an absolute reference standard. This provides a mutual check and validation of the uncertainties of CLARREO. However, CLARREO cannot independently provide a radiometric model for the Moon as CLARREO is not envisioned to continuously observe the Moon to account for its phase and libration variations.

## 3. Highlights From the Afternoon Discussion Groups

The workshop participants separated into three discussion groups: land variables, atmospheric variables, and clouds and oceans. Each group was charged with identifying critical climate variables susceptible to data gaps due to sensor-to-sensor bias variations, satellite sensor failure, or launch delay; proposing calibration techniques to ensure data continuity; and prioritizing strategies to maintain data records across gaps.

Drs. Changyong Cao of NOAA and Joseph Rice of NIST facilitated the general discussion during which each breakout group presented the results of their deliberations. Table 2 summarizes the critical climate variables identified by each group as well as each variable’s corresponding sensor types and required accuracies for climate monitoring. Speaking for the Land Variables Group, Dr. Kurtis Thome of NASA emphasized the importance of implementing high-accuracy calibrations for monitoring climate change, and suggested that establishing SI traceability in all measurement areas including pre-launch, onboard, and vicarious calibrations should be the primary strategy for effectively bridging data gaps. Thome noted that this effort should include the development of transfer standards that reduce the length of the calibration chain and lower final measurement uncertainties. Rice summarized the discussion of the Atmospheric Variables Group, which suggested mitigating data gaps by accessing data from other satellite sensors including non-US satellites or from targeted aircraft campaigns. The group also recommended better characterization of the atmosphere to improve upon the deficiencies in radiative-transfer models and increase the accuracy of aircraft campaigns. The discussion leader for the Clouds and Oceans Group, Dr. Robert Barnes of SAIC/NASA, echoed the findings of the other two groups, adding that the development of microwave standards is critical. Microwave measurements of sea surface temperature made by satellites are currently verified through comparison with measurements by buoys in the sea or by aircraft over-flight. In both cases, the uncertainties are larger than the requirements for monitoring the long-term climate change.

## 4. Summary of Recommended Strategies

Based on the reports from each discussion group, a list of strategies for managing gaps in the climate data record was compiled and is provided below. The strategies are grouped according to the kind of gap encountered. Data gaps can result from either a bias in the data acquisition due to different instruments or measurement methods or a disruption in data acquisition. Strategies for bridging a gap connect data across a gap for establishing trends, i.e., accounting for sensor-to-sensor bias variations. Strategies for mitigating a gap fill the gap using data acquired by alternative means to continuously provide critical information for people using satellite data.

The recommended strategies are varied and applicable to agencies, beyond NIST, that have space sensors providing Earth remote sensing data, such as NASA, NOAA, and USGS. The overarching recommendation, endorsed by all three breakout groups, is that all calibrations and measurement procedures for spaceborne and aircraft sensors should be rigorously traceable to the SI. Long-term monitoring of climate change will inevitably involve piecing together data from multiple sources. This process is made particularly robust when all measurements relevant to climate change are SI traceable to accepted physics-based, absolute scales. Such traceability is essential for bridging gaps across sensor bias variations, maintaining the integrity of the climate data record, and mitigating gaps due to lack of satellite data. The process of establishing SI traceability must begin in the sensor design phase and continue throughout pre-launch calibration and post-launch operation.

### 4.1 Strategies for Bridging a Gap

- *Pre-launch calibrations and onboard calibrations*—All spaceborne and aircraft sensors should be required to undergo thorough SI traceable calibrations prior to launch and on orbit. These calibrations should include component characterization and end-to-end system characterization of the spectral response of the sensor under operational scenarios to build a robust model of sensor performance and should follow the procedures outlined in “Best Practice Guidelines for Pre-launch Characterization and Calibration of Instruments for Passive Optical Remote Sensing” (NISTIR 7637). [7] Furthermore, a sensor-by-sensor study should be undertaken by respective agencies, before launch, with a risk analysis and impact study to outline strategies for overcoming gaps. Onboard calibration standards should be available for sensors to maintain SI traceable while on orbit.
- *Celestial standards*—High-resolution spectral calibrations of the Moon and stars, such as Vega, should be made to establish these celestial bodies as on-orbit, absolute reference standards for on-orbit characterization of instruments and could be used to determine any changes resulting from the launch of the instrument. The calibration of sensors against these standards should be integrated into the operational procedures of the sensors, and instrument designs should accommodate the use of celestial targets. For example, spacecraft in low Earth orbit require the ability to turn to view the Moon through their nadir-viewing optics.
- *Intercomparisons*—Procedures should be established to enable intercomparison of sensors between satellites, both US and non-US. This is crucial for monitoring and correcting measurement biases in instruments, which often change as the sensors age. Additionally, the community should support the activities of GSICS and encourage wider participation in GSICS across the world. GSICS promotes efforts to achieve the comparability of sensors on different satellites and to encourage pre-launch characterization and SI-traceable calibration.
- *Ground-based vicarious calibrations*—Ground sites should be further characterized to provide lowuncertainty, SI-traceable reference data for the calibration of space and aircraft sensors in the visible and reflected-solar infrared. Proper long-term maintenance of these ground sites should be considered.

### 4.2 Strategies for Mitigating a Gap

- *Airborne sensor campaigns*—Airborne sensor campaigns are a good alternative for collecting data during failures or launch delays of satellites sensors. The airborne sensors should be SI-traceable and calibrated in a manner consistent with their spaceborne counterparts. To improve the uncertainty of data from aircraft campaigns, there should be better characterization of the atmosphere to understand biases in radiative-transfer models. Airborne campaigns lack global coverage, but do provide reference data to aid data linkage across a gap. Agencies with capabilities to undertake airborne campaigns should ensure mission readiness if a data gap occurs and measurements are necessary to ensure climate data record continuity.
- *Non-US or other satellite assets*–The community should leverage its involvement in GSICS to acquire data from non-US or other satellite assets in the event of the failure of a particular satellite or sensor. These assets should be SI-traceable and calibrated.
- *Radiosonde measurements*—Data provided by in situ measurements such as radiosondes,^10^ for example through GRUAN (GCOS Reference Upper Air Network),^11^ are critical and should be considered in future plans for mitigating data gaps. Radiosondes measure the important atmospheric climate variables directly and can validate satellite observations. Effort should be made to improve the quality of these measurements and establish SI traceability.

## 5. Closing Remarks

Detecting the small signatures of climate change represents a formidable measurement challenge, requiring highly accurate and consistent measurements made over long periods of time. To minimize disruptions and maintain irrefutable climate records, participants at the workshop agreed that SI traceability in the remotesensing measurements for climate records is needed. Various strategies to ensure SI traceability on orbit and mitigate data gaps were recommended, and the need to coordinate across agencies to implement strategies was emphasized.

### 5.1 Opportunities for NIST to Contribute to the Implementation of the Recommended Strategies

Because of NIST’s role as a National Metrology Institute and its history as a collaborator in calibrating Earth remote sensing instruments, there was a natural progression in the discussion of strategies to recommend specific tasks to NIST. These are summarized below.

#### 5.1.1 Pre-Launch Calibrations and Onboard Calibrations

NIST should develop SI-traceable standards for microwave sensors to include brightness temperature standards and antenna characterization standards. These standards should have uncertainties that meet the requirements for calibrating the microwave sensors at low enough uncertainty to detect the signatures of climate change. The current lack of microwave standards hampers efforts to bridge data sets across a gap and ensure a continuous data set for monitoring climate change.

NIST should continue its efforts to improve prelaunch calibrations for space and aircraft sensors and disseminate those calibrations to the aerospace industry. NIST should also encourage SI traceability and promote implementation of the report, “Best practice guidelines for pre-launch characterization and calibration of instruments for passive optical remote sensing, NISTIR 7637.” [7] In particular, NIST should facilitate end-to end, system-level calibrations of sensors and actively deploy transfer standards, such as Travelling SIRCUS (Spectral Irradiance and Radiance Responsivity Calibrations using Uniform Sources) [10] and the Hyperspectral Image Projector (HIP) [11] for the reflected-solar region and the TXR for characterization of the emissivity and validation of the radiance of infrared sources used for calibrating space bound IR sensors. SIRCUS capabilities should be extended to longer wavelengths in the infrared as infrared laser technology advances.

NIST should facilitate improvement of on-board calibration. For instance, direct calibration of solar diffusers at NIST should be considered to shorten the measurement chain.

NIST should develop new on-board standards, such as stable lasers and laser diodes for space deployment. In particular, future CERES-type sensors would benefit from a temporally, spectrally, and spatially stable, high-accuracy lamp standard at 0.4 μm.

#### 5.1.2 Celestial Standards

NIST should implement the LUSI project to establish the Moon as an SI-traceable absolute radiometric standard to allow high accuracy radiometric calibration of sensors and long-term stability monitoring. This project should complement the NIST Stars program, which is developing a set of standard reference stars.

#### 5.1.3 Intercomparisons

NIST should aid the development of multiple independent calibration methods for cross checking sensors in orbit. In this regard, NIST should be involved in the characterization and calibration of ground sites for vicarious calibrations.

## Figures and Tables

**Table 1:** CLARREO’s science objectives determine which climate variables are monitored. The table below lists the type of feedback or response investigated by models for forecasting and their contributing climate variables. The types of sensors used to monitor the climate variables are also listed

Type of Feedback / Response	Climate Variables	Relevant Sensor Information
Cloud feedback and response:	Cloud fraction, height, temperature, visible optical depth, infrared emissivity, particle phase / size	Shortwave and longwave broadband radiative fluxes: Solar reflective and infrared spectra
Water vapor feedback and response:	Water vapor vertical profile	Infrared and solar reflective spectra
Lapse rate feedback:	Temperature vertical profile	Infrared spectra, GNSS-RO: Radio occultation observations using GPS signals
Snow / ice albedo feedback:	Snow / ice cover and albedo	Solar reflective spectra
Temperature response:	Temperature vertical profile	Infrared spectra, GNSS-RO
Greenhouse gas feedback:	Greenhouse gases	Infrared spectra

**Table 2:** Critical climate variables identified by the three breakout groups as susceptible to data gaps. The instruments and accuracy given as a relative uncertainty in percent or as an absolute uncertainty in K or W m^−2^ required to monitor each variable are also listed^a^,^c^

Classification	Variables	Instrument	Accuracy (*k* = 1)
Land	Vegetation	Vis radiometer	2 %
	Albedo	Vis radiometer	5 %
	Temperature	MW or IR radiometer	0.5 K
	Ozone	UV / Vis Spectrometer	3 %
	Water vapor	MW radiometer	1.0 K
		IR radiometer	1.0 K
Atmospheric	Precipitation	MW radiometer	1.25 K
	Clouds	Vis / IR radiometer	1 K
	Aerosols	Vis polarimeter	3 % (radiometric) 0.5 % (polarimetric)
	Greenhouse gases	IR radiometer	3 %
	TOA radiances	Broad band IR	1 W/m^2^
Clouds and oceans	Sea surface temperature	IR radiometer	0.1 K
		MW radiometer	0.03 K

a Reference [9]

b TOA: top of atmosphere; Vis: visible; MW: microwave; IR: infrared; and UV: ultraviolet.

## Footnotes

1 National Polar Orbiting Environmental Satellite System (NPOESS) is the next generation of low Earth orbiting environmental satellites. The program is managed by personnel from NOAA, NASA, and the Department of Defense. (Note: In February 2010, the U.S. Government cancelled the program and a restructuring is underway. For more information, see http://www.nasa.gov/pdf/441795main_GSFC_Roll_Out_Final.pdf.)

2 NPOESS Preparatory Project (NPP) aims to ensure the continuity of climate measurements between heritage sensors and the next generation sensors used by NPOESS.

3 Weng specifically alluded to the need to define a 3D antenna pattern (AP) at scan beam positions for accurately characterizing the main beam efficiency and side-lobe and development of an AP model that includes horn, cavity, reflector, polarizer grid, and side-lobe interaction with spacecraft.

4 MODIS is an advancement over heritage sensors like NOAA’s Advanced Very High Resolution Radiometer (AVHRR) and Landsat TM. It can monitor twenty bands in the solar reflective region of the spectrum spanning 0.4 μm to 2.2 μm and sixteen thermal emissive bands spanning the spectral region from 3.75 μm to 14.4 μm, providing information about land surface temperature and emissivity, land coverage, surface reflectance, and vegetation indices. The sensor is in a polar orbit onboard the Terra (EOS AM) and Aqua (EOS PM) satellites, and scans the whole Earth in two days. In addition to the onboard calibration features described above, MODIS also makes regular observations of deep space and the Moon to track the stability of the sensor in the solar reflective band.

5 NASA’s Earth Observing System (EOS) is a coordinated series of polar-orbiting and low inclination satellites for long-term global observations of the land surface, biosphere, solid Earth, atmosphere, and oceans. EOS is a major component of the Earth Science Division of NASA’s Science Mission Directorate. For more information, see http://eospso.gsfc.nasa.gov/.

6 The system-level VIIRS characterization has begun with NIST participation at the contractor’s (Ball Aerospace) site in Boulder, CO and is currently in progress.

7 The NRC’s report defined CLARREO as two parts. NOAA is responsible for continuing the long-term records of the radiation budget and maintaining the satellite sensors, Clouds and Earth’s Radiative Energy System (CERES) and Total Solar Irradiance Sensor (TSIS). NASA is responsible for measuring the Earth’s reflected solar and infrared spectra at high spectral resolution and very low radiometric uncertainty. NASA is also charged with collecting on-board Global Positioning System (GPS) radio occultation measurements to deduce atmospheric refractivity, density, pressure, and temperature profiles with a very high degree of accuracy.

8 Typical solar attenuators are a solar diffuser panels or apertures. They are used to calibrate the sensor on-orbit. The sun is the radiation source for the calibration; however, it is attenuated to the radiance level of the Earth.

9 The point is that satellite sensors unable to view the Moon can be referenced to the Moon by using a simultaneous nadir overlap (SNO) against a satellite that can view the Moon as a reference standard.

10 A radiosonde is a unit carried by a weather balloon to record and transmit direct measurement of atmospheric variables, such as temperature, relative humidity, pressure and wind speed, as a function height and its global location tracked by radar or using GPS (Global Positioning Service). They are not optical radiometric sensors such as those deployed on satellites. However, they are launched around the world from various weather stations and provide valuable in situ data for numerical models for weather prediction.

11 For more details regarding GRUAN, see http://www.wmo.int/pages/prog/gcos/index.php?name=GRUAN. The Global Climate Observing System (GCOS) is a joint undertaking of World Meteorological Organization (WMO) and other international organizations. For more information about GCOS, see http://www.wmo.int/pages/prog/gcos/index.php?name=AboutGCOS.

## 6. Appendix A: Agenda

Introduction, Welcome, and Workshop Goals

   Dr. Raju Datla, NIST

   Dr. Katharine Gebbie, Director, Physics

   Laboratory, NIST

   Dr. Gerald Fraser, Chief, Optical Technology

   Division, NIST

Session 1: Invited Talks

“Climate variables and prioritization for climate data records”

   Dr. Bruce Wielicki, NASA

“GSICS and NIST role”

   Dr. Mitch Goldberg, NOAA

“Microwave data and need for NIST support”

   Dr. Fuzhong Weng, NOAA

“MODIS lessons: NIST role in sensor calibration and inter-comparison”

   Dr. Xiaoxiong (Jack) Xiong, NASA

“CLARREO goals and NIST support”

   Dr. Martin Mlynczak, NASA

“LUSI—the Moon as an absolute reference standard”

   Dr. Thomas Stone, USGS

“Operational climate program at NCDC”

   Dr. John Bates, NOAA

“NOAA/NIST collaboration to support climate research”

   Dr. Al Powell, NOAA

Session 2: Discussion Groups

Group 1: Land variables

   Discussion leader—Dr. Kurt Thome, NASA

Group 2: Atmospheric variables

   Discussion leader - Dr. Joseph Rice, NIST

Group 3: Clouds, Oceans, etc.

   Discussion leader—Dr. Robert Barnes, SAIC/NASA

Session 3: Reports From Discussion Groups

   Facilitators—Dr. Joseph Rice, NIST and Dr. Changyong Cao, NOAA

Closing

   Dr. Raju Datla, NIST

## 7. Appendix B: Participants

Harvard University

   John Dykema

NASA and NASA Contractors

   Robert Barnes, Rosemary Baize, James Butler, Martin Mlynczak, Kory Priestly, Paul Racette, Kurt Thome, Kevin Turpie, Bruce Wielicki, Xiaoxiong (Jack) Xiong, and David Young

NIST

   Steven Brown, Catherine Cooksey, Amanda Cox, Raju Datla, Gerald Fraser, Katharine Gebbie, Leonard Hanssen, Heather Patrick, Carol Johnson, Sergey Mekhontsev, Joseph Rice, Eric Shirley, Allan Smith, and David Walker

NOAA

   John Bates, Changyong Cao, Lawrence Flynn, Mitch Goldberg, Andrew Heidinger, Alex Ignatov, Mike Ondrusek, George Ohring, Al Powell, Menghua Wang, Fuzhong Weng, Xiangqian Wu, Yunyue Yu, Xuepeng Zhao, and Cheng-Zhi Zou

University of Wisconsin

   Bob Knuteson and Dan Laporte

USGS

   Tom Stone

Utah State University

   Harri Latvakoski and Deron Scott.
